# Supplementary material for: Directional Submicrofiber Hydrogel Composite Scaffolds Supporting Neuron Differentiation and Enabling Neurite Alignment
Source: Int J Mol Sci. 2022 Sep 29;23(19):11525. doi: 10.3390/ijms231911525 (PMC9569964; doi:10.3390/ijms231911525)

Single channels of confocal images for random and aligned scaffolds at day 1 and 14

ALIGNED

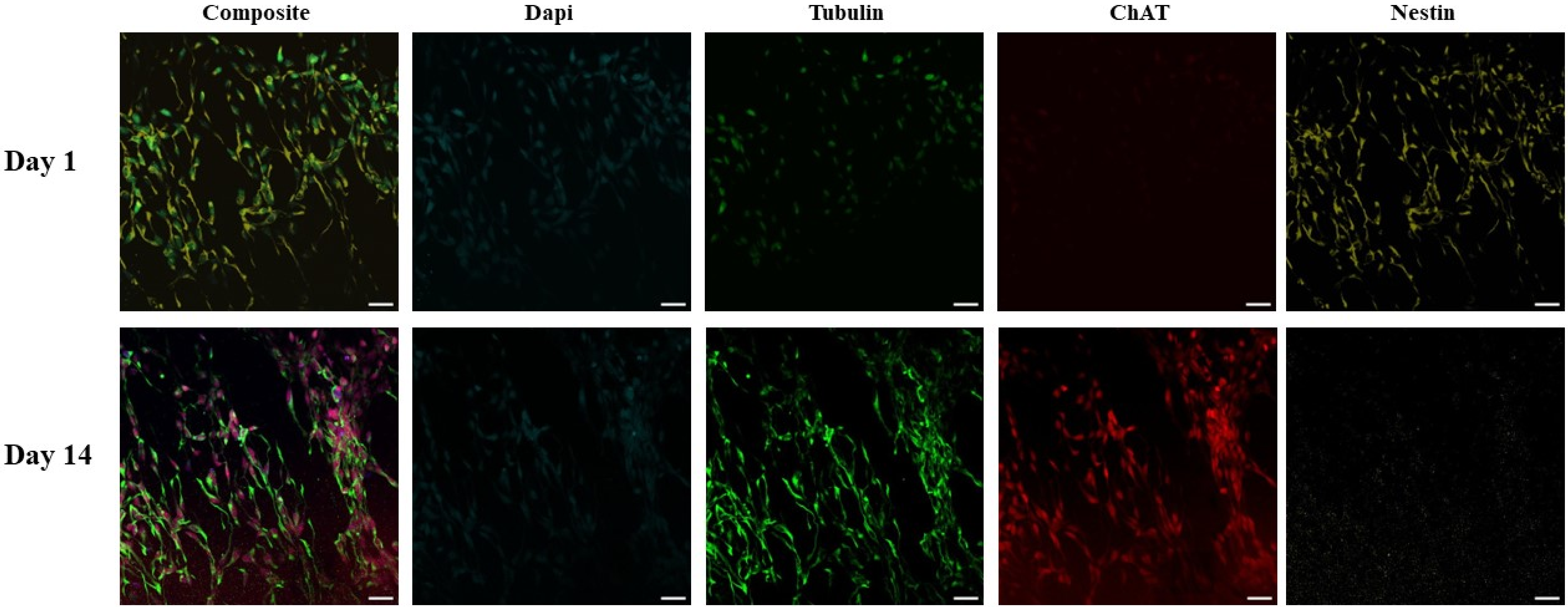

Supplement: Supplementary file 1 [file ijms-23-11525-s001.zip › SI1_Aligned scaffolds - day 1 and 14.pdf]
