# Supplementary figures and images for: Directional Submicrofiber Hydrogel Composite Scaffolds Supporting Neuron Differentiation and Enabling Neurite Alignment
Source: Int J Mol Sci. 2022 Sep 29;23(19):11525. doi: 10.3390/ijms231911525 (PMC9569964; doi:10.3390/ijms231911525)

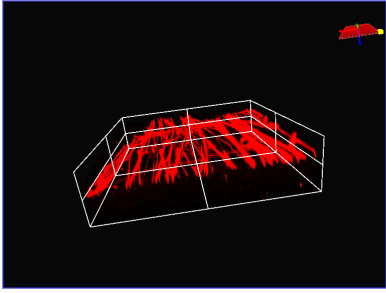

Supplement: Supplementary file 1 [file ijms-23-11525-s001.zip › SI3_only fibers 10x_0011.pdf]
